# Numerical investigation of dynamic responses and mooring forces of submerged floating tunnel driven by surface waves

**DOI:** 10.1038/s41598-020-75907-8

**Published:** 2020-11-02

**Authors:** Xuebin Chen, Zhiwu Chen, Shuqun Cai, Wei Xu, Xianrong Zhuo, Jiangen Lv, Jiajian Zhao

**Affiliations:** 1 College of Urban and Rural Construction, Zhongkai University of Agriculture and Engineering, Guangzhou, 510225 China; 2 State Key Laboratory of Tropical Oceanography, South China Sea Institute of Oceanology, Chinese Academy of Sciences, Guangzhou, 510301 China; 3 Department of Physics, Hong Kong University of Science and Technology, Clear Water Bay, Kowloon, Hong Kong China

**Keywords:** Hydrology, Ocean sciences

## Abstract

Based on Navier–Stokes equations, a numerical model for studying the dynamic responses and mooring forces of the moored Submerged Floating Tunnel (SFT) driven by surface waves is presented in this paper. The mechanics models of the vertically and inclinedly moored floating body under wave forces are built, and the overset meshing method is employed to dynamically configure the computational meshes. Two laboratory experiments are used for validating the numerical model in terms of motion responses and mooring forces of the SFT, indicating the proposed model is capable of accurately simulating the instantaneous position of the body under the wave action. This hydrodynamic model is then utilized to simulate the wave–structure interaction of the prototype SFT designed for Qiongzhou Strait located between Mainland China and Hainan Island. The effects of the fundamental structure parameter, or the inclined mooring angle (IMA), on the dynamic responses of SFT are analyzed. The numerical experiments not only shed light on the mooring forces, as well as pitch, sway and heave responses of the SFT with various values of IMA, but also provide guidance for the choice of IMA in engineering design. The range of IMA is separated into five zones, and Zone 2 is regarded as the best choice for the design of IMA for both motion displacements and mooring forces are relatively small in this zone. Zone 3 is considered to be the worst choice as not only are motion responses of SFT severe in this zone, but also the mooring chains are at the risk of going slack under severe wave conditions.

## Introduction

**Figure 1:**
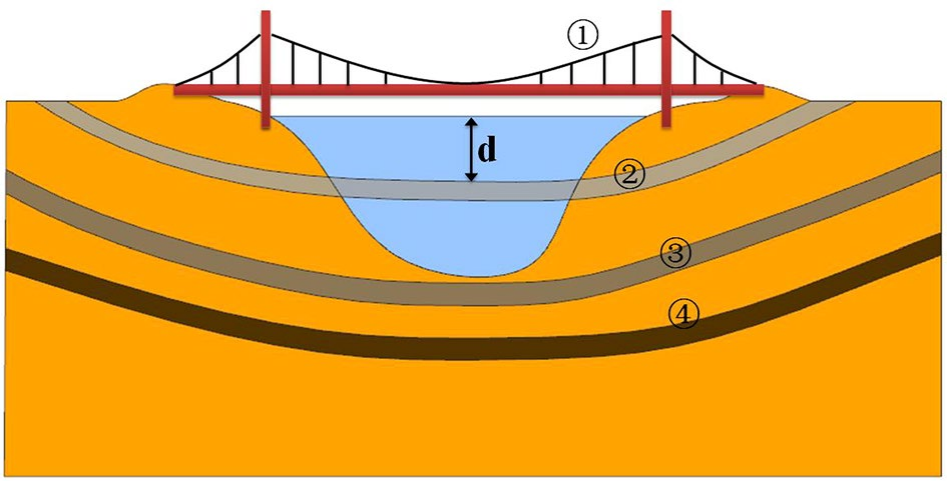
Crossing method on the channel [(1) bridge, (2) submerged floating tunnel, (3) immersed tunnel, and (4) submarine tunnel)].

Recently, the interest in Submerged Floating Tunnel (SFT), which is also named Archimedes Bridge, has increased as coastal and ocean engineers search for an innovative transportation concept for crossing the deep strait, lake, fjord and other deep and long waterways^1–9^. The characteristics of SFT are not burying under or putting upon waterbed but a tube-like floating tunnel at a certain water depth supported by its self-buoyancy and constrained by a mooring system anchored to the waterbed. Figure 1 presents four common methods of crossing strait or river, i.e., bridge, SFT, immersed tunnel and submarine tunnel. SFT has an advantage over the other three methods in special water areas, such as deep sea, long span, environmental sensitivity^2,4,10^. Although SFT has been proposed as an effective traffic solution to cross waterways, such as the Strait of Messina in Italy, Hogsfjord in Norway, South Sea of Korea, Funka Bay in Japan, Qiongzhou Strait in China, Bohai Strait and Taiwan Strait in China^4,10–15^, it has yet to be realized due to a lack of safety assurance. For an SFT to be safe during its whole life, it is important to develop efficient and accurate numerical models to determine the hydrodynamic loads on the SFT and its potential motions in response to extreme wave conditions.

Over the years, extensive studies on nonlinear wave–structure interactions have been conducted and numerous conclusions have been made based on both experimental and numerical methods in the design of SFT and other floating structures. In laboratory experiments, many monitoring results are gained by laboratory instruments, such as wave forces^16,17^, motion responses^18–21^, mooring forces^22–26^, velocity fields^27,28^ and so on. These experiments not only shed light on the influences of hydrodynamic and structure parameters on motion responses as well as forces acting on the small size structures, but also provide guidance for the protection and design of the structure. Assuming the flow was non-viscous and irrotational, earlier studies mainly used the potential flow theory to simulate the dynamics of the floating body. Based on potential theory, Koo and Kim^29,30^ built a 2D numerical wave tank to study the fierce interactions between stationary or freely floating bodies and surface waves. Kunisu^31^ also utilized Morison’s equation based on potential theory to evaluate the wave forces on SFT, and the results indicated that inertia force and drag force simultaneously worked on the structure. Guerber et al*.*^32^ successfully used the fully non-linear potential flow (FNPT) model to simulate the fully nonlinear interaction of waves with a submerged horizontal cylinder. Subsequently, the model in Guerber et al*.*^32^ was extended by Dombre et al*.*^33^ to solve freely floating bodies on the free surface, and the accuracy and convergence of the extended model in solving wave–structure interactions were demonstrated. Nevertheless, as the convection potential flow method assumes an irrotational flow, the nonlinear interactions of waves with obstacles, including flow separation, vortex generation, and shedding, are difficult to determine satisfactorily. Besides, this type of model is not capable of predicting the pitch motion of an SFT caused by viscous damping in the vicinity of the structure, although a good agreement of the sway and heave motions are gained.

With the development of computer technology over past decades, the latest computers are now capable of carrying out a direct numerical simulation of the wave–structure interaction based on the Navier–Stokes (N–S) models^34–39^. Most of these studies listed above only dealt with the interactions between water waves and fixed structures without considering the influence of motion response on wave–structure interaction. One of the key issues for solving the N–S equations in the treatment of the varying positions of the structure during the interaction is how to deal with the body motion in the computation. Because the body surface is a part of the boundary of the fluid domain, it is necessary to update the computation mesh according to the geometry change in the fluid domain resulting from the body motion. A mesh deforming method^40^ is commonly used to handle the mesh update, but mesh quality might be reduced severely when the body undergoes large motions^41^. The method of adaptive mesh refinement^42^ is suitable for arbitrary motions of a body, while the calculation is time-consuming and mesh quality is unable to be guaranteed in the long period of body motion^43^. A layering mesh method based on CFD software package, Fluent, has been successfully developed to study the interaction between bridge decks and incident waves by User Defined Function^44,45^, while the structure is only allowed to move in one direction. An efficient method for preserving good mesh quality in the simulation of body motion, especially large body movements in a long duration, is the dynamic overset mesh technique, which has been successfully applied to practical hydrodynamic problems involving structure–wave interactions^46,47^.

In order to protect the floating structure, tensioned mooring systems are often used to restrain the motion of the body, and an accurate prediction of the mooring forces, as well as motion responses, are critically important for the structure design. As mentioned above, numerical methods based on potential theory are inadequate for the prediction of the multiple motions and velocity field around the body, researchers are using the N–S model to predict the instantaneous nonlinear interaction. Rahman et al*.*^22^ successfully developed a porous body model coupled with the Volume of Fluid (VOF) method to predict the wave deformation and dynamics of the SFT in a small size, while the model suffered from high computational cost in the grid generation and re-meshing. Subsequently, Peng et al*.*^24^ applied the Immersed Boundary Method (IBM) to deal with the movable SFT under waves, and the numerical model based on Eulerian grid methods had difficulties in treating structures with complex geometries. Ren et al*.*^48^ applied a Smoothed Particle Hydrodynamics (SPH) model to simulate the nonlinear interaction between waves and the SFT, a good agreement was gained between numerical results and experimental data except for the prediction of the transmitted wave with a maximum under-prediction of 40%, which was correlated with the artificial viscosity used in momentum equations.

So far, as for such a novel SFT, researchers are still searching for an efficient method to accurately predict the instantaneous wave–structure interaction. Besides, researches on the performance of a prototype SFT in severe wave conditions are still scarce at present. Hence, the objective of this paper is to first develop an efficient method based on the N–S model to simulate the interaction between incident surface wave and moored submerged floating tunnel, then analyze and quantify the performance of a moored prototype SFT under severe wave conditions. The CFD software package, FLUENT 17.0, is utilized as a basic solver in this model, and User Defined Function (UDF) written in C programming language is incorporated into the model to solve the motion equations and control the body motion. The overset method is utilized to handle the mesh updating, which is supported by the software package. This paper is organized as follows. “Numerical model” presents a description of the methodology used in this paper focusing on the mechanics of the vertically and inclinedly moored floating tunnel. In “Model verification and discussions”, two experimental results are used for validating the numerical model in terms of motion responses and mooring forces of the SFT. In “Hydrodynamic response of a submerged floating tunnel”, the interaction between waves and the prototype SFT planed across Qiongzhou strait, China, is simulated. Finally, “Summary and conclusions” summarizes the findings with several conclusions.

## Numerical model

### Governing equations

**Figure 2:**
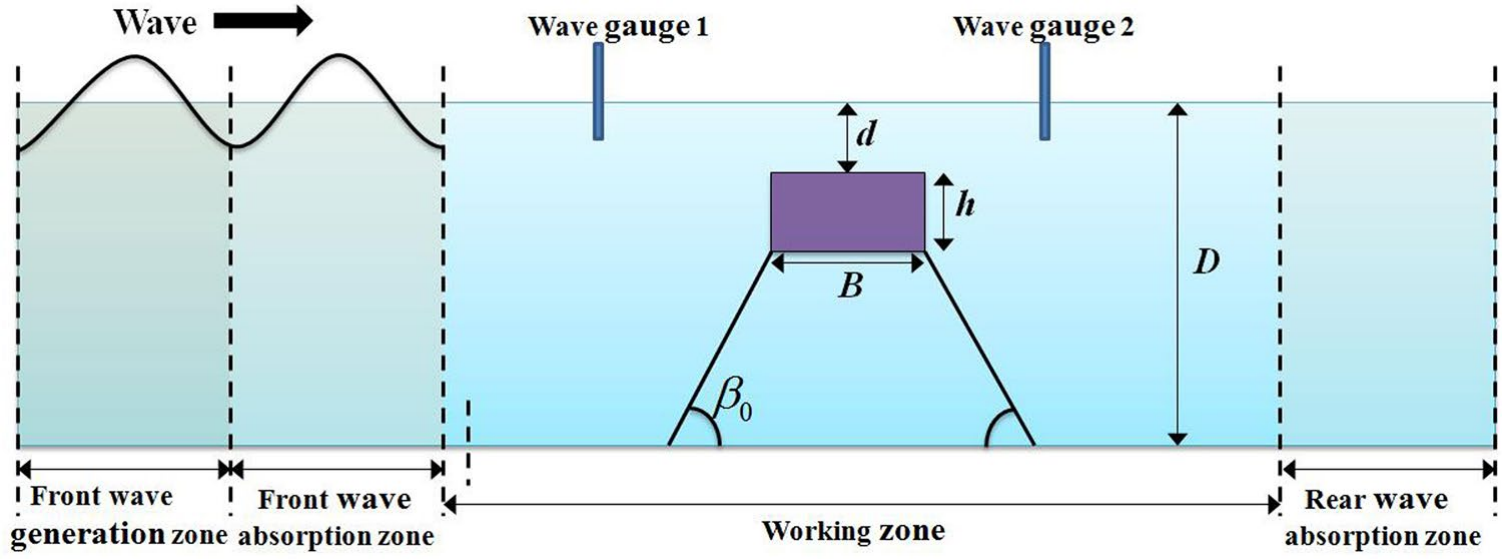
Schematic of the computational domain for a submerged floating body under the regular wave.

In this study, the employed numerical wave tank used to simulate the interaction between submerged floating body and incident wave is based on the N–S solver. The flow of an incompressible viscous fluid, as shown in Fig. 2, is governed by the continuity equation and momentum equations:

1 $$ \frac{{\partial \left( {\rho u} \right)}}{\partial x} + \frac{{\partial \left( {\rho v} \right)}}{\partial y} = 0 $$

2 $$ { }\frac{{\partial \left( {\rho u} \right)}}{\partial t} + u\frac{{\partial \left( {\rho u} \right)}}{\partial x} + v\frac{{\partial \left( {\rho u} \right)}}{\partial y} = - \frac{\partial p}{{\partial y}} + \mu \left( {\frac{{\partial^{2} u}}{{\partial x^{2} }} + \frac{{\partial^{2} u}}{{\partial y^{2} }}} \right) + S_{x} $$

3 $$ { }\frac{{\partial \left( {\rho v} \right)}}{\partial t} + u\frac{{\partial \left( {\rho v} \right)}}{\partial x} + v\frac{{\partial \left( {\rho v} \right)}}{\partial y} = - \frac{\partial p}{{\partial y}} - \rho g + \mu \left( {\frac{{\partial^{2} v}}{{\partial x^{2} }} + \frac{{\partial^{2} v}}{{\partial y^{2} }}} \right) + S_{y} $$

where $\rho$ is the mass density of the air phase or water phase; *u* and *v* are the velocity components in the *x*-direction and the *y*-direction, respectively; *g* is the gravitational acceleration; $\mu$ is the fluid viscosity; *p* is the fluid pressure;${ }S_{x}$ and $S_{y}$ are the momentum sources in the *x*-direction and *y*-direction, respectively.

### Numerical wave tank

The schema of a numerical wave tank is shown in Fig. 1. The numerical wave tank is divided into four zones: front wave generation zone, front wave absorption zone, working zone, and rear wave absorption zone. The front wave absorption zone is utilized to eliminate the reflected wave from the working zone in case of affecting the wave generation zone. A flow relaxation scheme is adopted at the front wave generation zone and front wave absorption zone. The relaxation method updates the interior solution *u*_*c*_ and *v*_*c*_ in the relaxation zones as

4 $$ u_{r} = C\left( x \right)u_{c} + \left[ {1 - C\left( x \right)} \right]u_{i} ,\;v_{r} = C\left( x \right)v_{c} + \left[ {1 - C\left( x \right)} \right]v_{i} $$

where subscript *r* stands for the relaxed solution value, subscript $c$ stands for the interior computed value, and subscript *i* stands for the predefined incident value. $C\left(x\right)$ is a function varying with $x$, where $x=0$ represents the start of front wave generation zone and front wave absorption zone, $x=1$ represents the end of front wave generation zone and front wave absorption zone. The values of ${C}_{fg}\left(x\right)$ in the front wave generation zone and ${C}_{fa}\left(x\right)$ in front wave absorption zone are defined in Eqs. (5) and (6), respectively.

5 $$ C_{fg} \left( x \right) = \cos \left( {\frac{\pi }{2}x} \right) $$

6 $$ C_{fa} \left( x \right) = \sin \left( {\frac{\pi }{2}x} \right) $$

**Figure 3:**
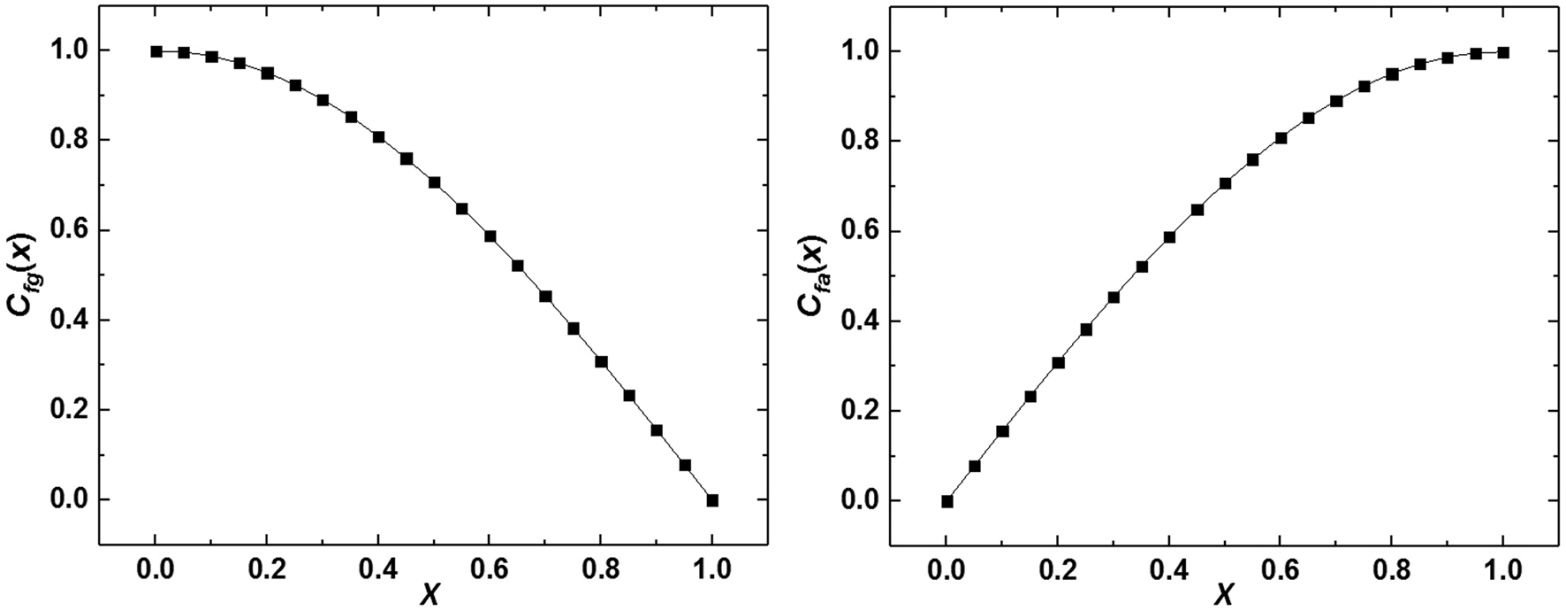
The variation of *C*(*x*) with respect to *x*. (**a**) Front wave generation zone; (**b**) front wave absorption zone.

In the front wave generation zone, $C\left(0\right)=1$ and $C\left(1\right)=0$; In the front wave absorption zone, $C\left(0\right)=0$ and $C\left(1\right)=1$. According to Eqs. (5–6), Fig. 3 shows the variation of *C*(*x*) with respect to *x* in the front wave generation zone and front wave absorption zone. As the relaxation scheme is utilized in the front wave generation zone and front wave absorption zone in the flow, momentum sources ${S}_{x}$ and ${S}_{y}$ described in Eqs. (1–2) are determined by the following equations:

7 $$ \begin{aligned} S_{x} {\kern 1pt} {\kern 1pt} = & \frac{\rho }{\Delta t}(C - 1)(u_{c}^{n} - u_{i}^{n} ) + \rho (1 - C)\left( {\frac{1}{\rho }\frac{{\partial p_{c} }}{\partial x} + \frac{{\partial u_{i} }}{\partial t} + u_{i} \frac{{\partial u_{i} }}{\partial x} + v_{i} \frac{{\partial v_{i} }}{\partial y}} \right) \\ & + (1 - C^{2} )\left( {u_{i} \frac{{\partial u_{i} }}{\partial x} + v_{i} \frac{{\partial u_{i} }}{\partial y}} \right) + C(1 - C)\left[ {\left( {u_{c} \left( {\frac{{\partial u_{i} }}{\partial x} + v_{c} \frac{{\partial u_{c} }}{\partial y}} \right)} \right.} \right. + \left. {\left. {\left( {u_{i} \frac{{\partial u_{c} }}{\partial x} + v_{c} \frac{{\partial u_{c} }}{\partial y}} \right)} \right]} \right\}\; \\ & - \rho \{ (C^{2} - 1)\left( {u_{c} \frac{{\partial u_{c} }}{\partial x} + v_{c} \frac{{\partial u_{c} }}{\partial y}} \right) \\ \end{aligned} $$

8 $$ \begin{aligned} S_{y} {\kern 1pt} {\kern 1pt} = & \frac{\rho }{\Delta t}(C - 1)(v_{c}^{n} - v_{i}^{n} ) + \rho (1 - C)\left( {\frac{1}{\rho }\frac{{\partial p_{c} }}{\partial y} + \frac{{\partial v_{i} }}{\partial t} + u_{i} \frac{{\partial v_{i} }}{\partial x} + v_{i} \frac{{\partial v_{i} }}{\partial y} + g} \right) \\ & + (1 - C^{2} )\left( {u_{i} \frac{{\partial v_{i} }}{\partial x} + v_{i} \frac{{\partial v_{i} }}{\partial y}} \right) + C(1 - C)\left[ {\left( {u_{c} \left( {\frac{{\partial v_{i} }}{\partial x} + v_{c} \frac{{\partial v_{i} }}{\partial y}} \right)} \right.} \right. + \left. {\left. {\left( {u_{i} \frac{{\partial v_{c} }}{\partial x} + v_{i} \frac{{\partial v_{c} }}{\partial y}} \right)} \right]} \right\} \\ & - \rho \{ (C^{2} - 1)\left( {u_{c} \frac{{\partial v_{c} }}{\partial x} + v_{c} \frac{{\partial v_{c} }}{\partial y}} \right){\kern 1pt} \\ \end{aligned} $$

To avoid wave reflection from the outlet boundary, one porous wave absorber is prescribed at the outlet to absorb wave energy before reflection occurs^49^. The porous media method developed in the present work is a simulation of the physical energy dissipation mechanism. For this aim, the momentum source term composed of a viscous dissipation term and an inertial term is added to the Navier–Stokes equations as follows:

9 $$ S_{i} = - \left( {\frac{\mu }{\alpha }{ }v_{i} + C_{2} { }\frac{1}{2}\rho \left| v \right|v_{i} } \right){ } $$

where $$ S_{i}$$ is the source term for the *i*th momentum equation, $$\left| v \right|$$ is the magnitude of the velocity, $$\alpha$$ is the permeability and $$C_{2}$$ is the inertial resistance factor. In the current work, the wave energy could be dissipated effectively using the viscous dissipation term alone and therefore $$C_{2}$$ is set to zero. To avoid the abrupt change of the flow resistance in the wave absorption region, the value of $$1/\alpha $$, which is called the viscous resistance coefficient, is set up to increase the linearly in this region:

10 $$ { }\left( {1/\alpha } \right)_{i} = 10^{6} \frac{{x_{i} - x_{0} }}{{x_{e} - x_{0} }}{ },{ }x_{0} < x_{i} < x_{e} $$

where ${x}_{0}$ and ${x}_{e}$ are the $x$ coordinates of the two end points of the rear wave absorption region. Usually, the length of the wave absorption region is set to be two wavelengths.

By incorporating the momentum source defined by UDF into the CFD solver, we can model the numerical wave tank in the present study. Further information about the validation of the numerical wave tank can be found in Chen et al*.*^50^.

### Mechanics model of the floating body

**Figure 4:**
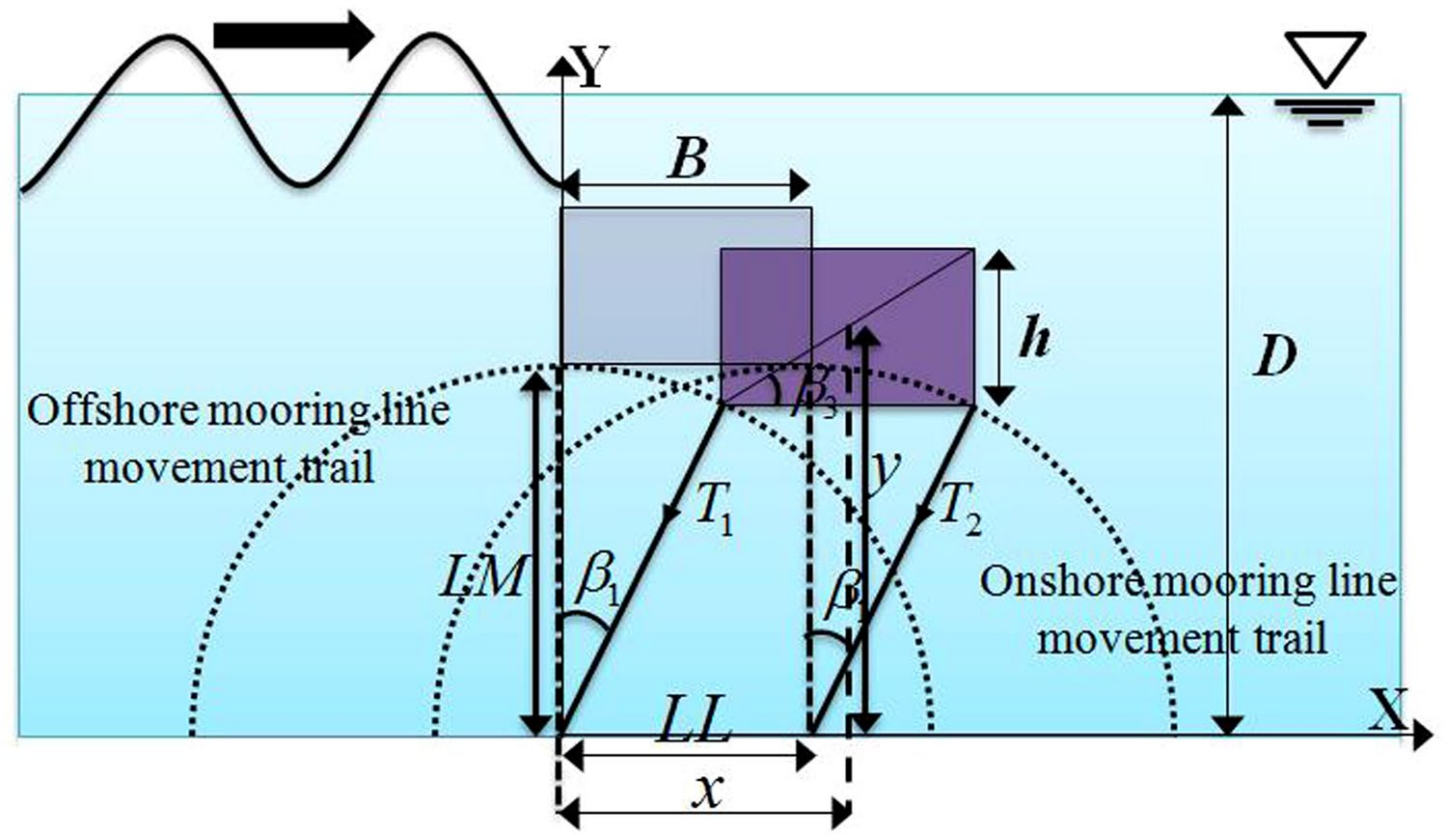
Instantaneous position of the vertically moored floating body during its interaction with a wave.

The dynamic motions of the floating body are driven by incident waves in combination with mooring chains. Thus, the correlation between forces acting on the moored body and motions of the moored body should be gained to establish the mechanics model of the floating body. The instantaneous position of the vertically moored floating body during its interaction with a wave is shown in Fig. 4. It is assumed that the weight of the floating body is light compared to the buoyancy forces acting on it vertically, resulting in no slack state in the mooring lines with no impulsive force on it. This also causes only sway and heave motions of the body and no rotational motion. Based on the above considerations, the dynamics of the floating body are calculated using the following equations. Defining the mass of the body as $m$, horizontal and vertical acceleration of the body as ${a}_{x}$ and ${a}_{y}$, respectively, the resultant horizontal and vertical force acting on the body as ${F}_{x}$ and ${F}_{y}$, respectively, we get,

11 $$ F_{x} = F_{wave force,x} + T_{mooring force,x} = ma_{x} $$

12 $$ F_{y} = F_{wave force,y} + T_{mooring force,y} + mg = ma_{y} $$

where ${F}_{wave force,x}$ and ${T}_{mooring force,x}$ are the horizontal components of wave forces and tensile forces acting on the mooring lines, respectively;${F}_{wave force,y}$ and ${T}_{mooring force,y}$ are the vertical components of wave forces and tensile forces acting on the mooring lines, respectively. The wave forces acting on the surfaces of the submerged body can be calculated by integrating the pressure and shear stress on its surface.

Furthermore, as the vertically moored floating body does not rotate during its interaction with incident wave, the summation of the moments acting on its center of gravity should be zero, i.e.,

13 $$ M_{cg} = M_{wave force} + M_{mooring force} = 0 $$

where $M_{cg}$ represent total moments acting on the body; $M_{wave force}$ and $M_{mooring force}$ are the moments induced by wave forces and tensile forces, respectively. Also, referring to Fig. 4, considering no slack state, the geometry of the mooring line gives the following equation.

14 $$ LM^{2} = \left[ {x - \frac{h}{{2tan\beta_{3} }}} \right]^{2} + \left[ {y - \frac{h}{2}} \right]^{2} $$

where *LM* is the length of the mooring line and $h$ is the thickness of the floating body; $x$ is the distance along x-direction between the offshore anchoring point and gravity center of the floating body.

**Figure 5:**
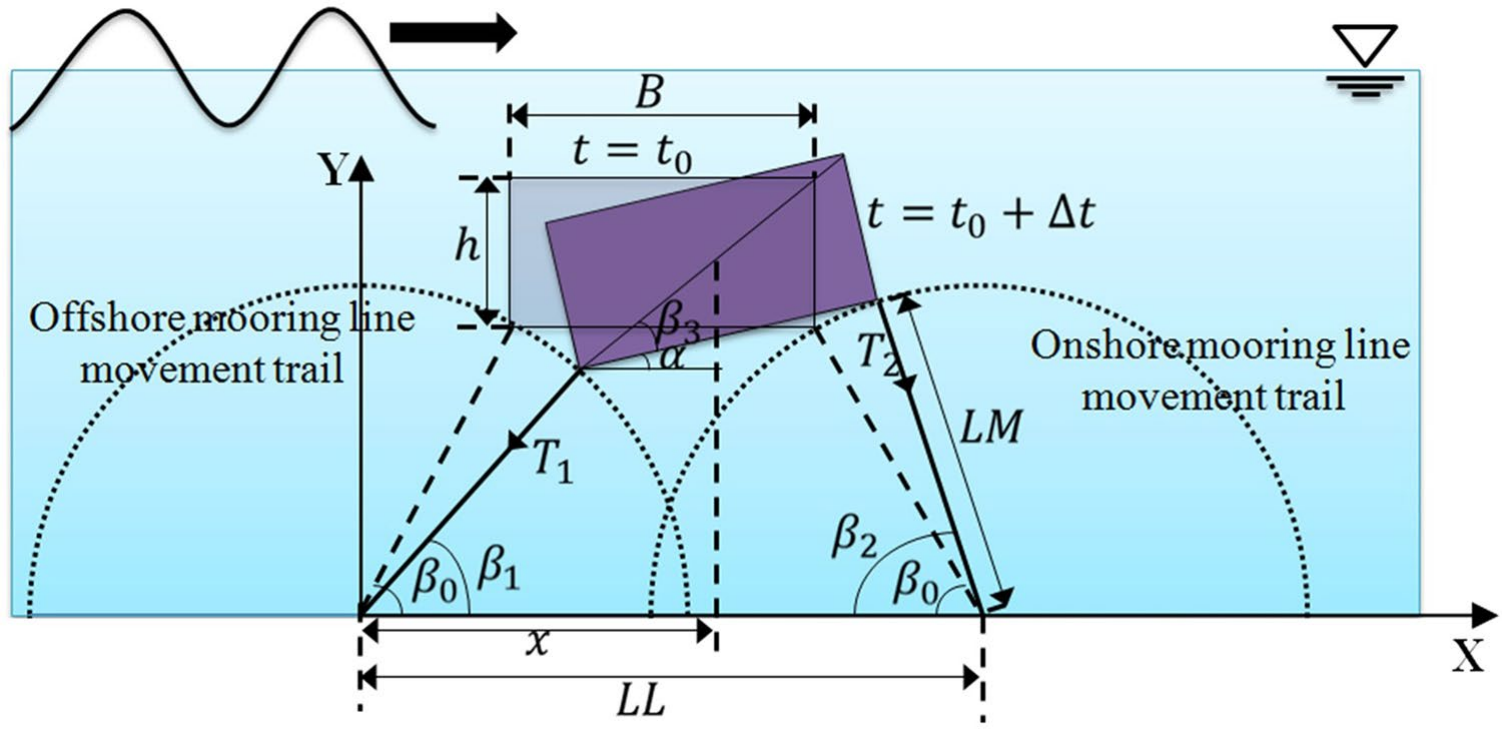
Instantaneous positions of the inclinedly moored floating body during its interaction with a wave.

On the other hand, when the body is anchored to the bottom of the wave tank with inclinedly aligned mooring lines, the dynamics of the floating body at any instantaneous position is shown in Fig. 5, where the floating body has three degrees of freedom driven by incident waves, i.e., the pitch motion, the sway motion, and the heave motion. Defining the mass moment of inertial of the floating body as $I$, the acceleration of pitch motion as ${\alpha }_{body}$, the summation of the moments acting on its center of gravity should be, i.e.,

15 $$ M_{cg} = M_{wave force} + M_{mooring force} = I\alpha_{body} $$

Considering the no-slack condition of the mooring lines, the geometric feature of the wave–structure interaction in Fig. 5 gives the following additional equations:

16 $$ LM^{2} = \left[ {x - \frac{h}{{2sin\beta_{3} }}\cos \left( {\beta_{3} + \alpha } \right)]^{2} + } \right[y - \frac{h}{{2sin\beta_{3} }}\sin \left( {\beta_{3} + \alpha } \right)]^{2} $$

17 $$ LM^{2} = \left[ {LL - x - \frac{h}{{2sin\beta_{3} }}\cos \left( {\beta_{3} - \alpha } \right)]^{2} + } \right[y - \frac{h}{{2sin\beta_{3} }}\sin \left( {\beta_{3} - \alpha } \right)]^{2} $$

### Mesh update method

**Figure 6:**
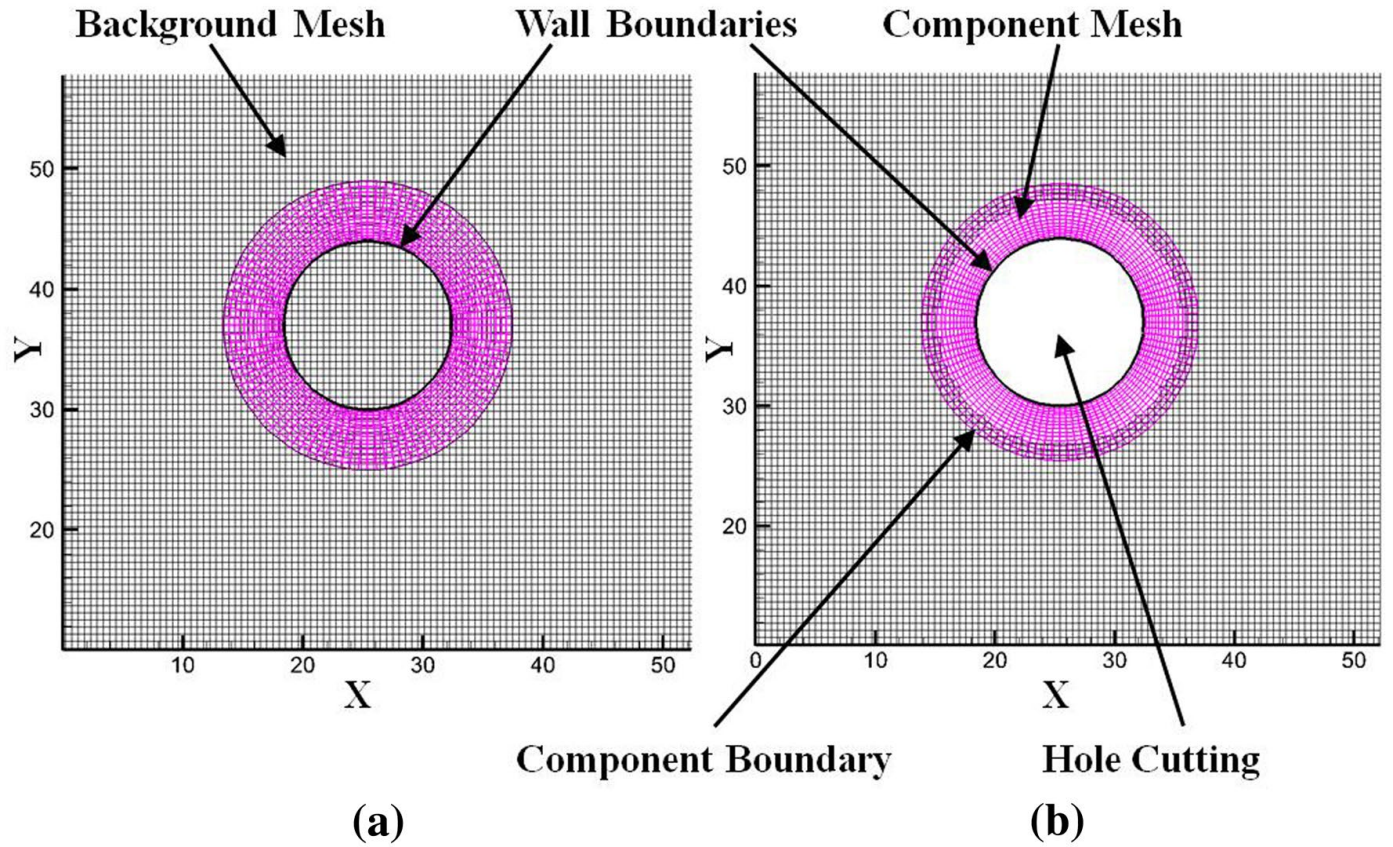
Schematic drawing of two cell zones before and after hole cutting. (**a**) Overlapping cell zones before hole cutting; (**b**) extracted domain after hole cutting.

The overset mesh, also known as Chimera or overlapping mesh, is used to facilitate the motions of the floating body due to the incident waves in this study. Compared with traditional rigid and deforming mesh options in the software package, the overset mesh is a very effective way of preserving high mesh quality in the process of fierce fluid–structure interaction with large motion displacements. In the overset meshing methodology, two regions, background and component meshes are created. The meshes in each cell zones are independent as these cell zones are not forced to be aligned with each other. In the process of hole cutting, the cells at the overlapping zones are deactivated, and different cell zones are coupled by an interface to provide continuous solutions. The deactivated cells in the hole cutting are called the dead cells. The donors are the cells that supply flow information to the associated overlapping cell zones. Receptors obtain information from the donors in the corresponding overlap cell zone and vice-versa^51^. A schematic drawing of overlapping component and background mesh zones is shown in Fig. 6a, and the extracted flow domain after overset hole cutting is shown in Fig. 6b.

The overset meshes move together with the floating body, thus no mesh deformation exists in the duration of motion responses. The background meshes keep stationary in space and the overset meshes are free to move as long as the cells in the overlap zones work well. The cells in the overlapping region are solved simultaneously by the discretized governing equations on the overset mesh boundaries. Thus, the coupling between the meshes is implicit. In the interpolation method, receptor cells at the overset interface obtain messages from nearby donors, which can be interpolated by the following equation^51^.

18 $$ \emptyset ^{h} = \mathop \sum \limits_{i = 0}^{{N_{d} }} w^{i} \emptyset^{i} $$

where $\emptyset$ is the solution variable, $w$ is the interpolation weights and $N_{d}$ is the number of donors.

### Numerical implementation

In the solver, the governing equations are discretized based on the finite volume method (FVM). The Second Order Upwind algorithm is used to discretize the momentum convection. Coupled algorithm is used for the pressure–velocity coupling with Pressure Staggering Option (PRESTO) discretization scheme. The Volume of Fluid (VOF) method is adopted to capture the fluctuating water surface. The laminar model is used to simulate the incompressible and viscous flow.

The procedure for solving the interaction between wave and moored floating body is described as follows. At the very beginning of every time step, the flow field is solved by the main program firstly. Then the wave forces and moments are obtained by using the User-Defined Functions with an incorporated C programming language to integrate the pressure and shear stress over the floating body. For the vertically moored floating body, Eqs. (11–13) are then solved simultaneously to get the values of above unknown parameters (i.e., ${a}_{x}$,${a}_{y}$,${T}_{1}$ and ${T}_{2}$). For the inclindely moored floating body, Eqs. (11, 12, 15–17) are then solved simultaneously to get the values of above unknown parameters (i.e., ${a}_{x}$,${a}_{y}$,${\alpha }_{body}$,${T}_{1}$ and ${T}_{2}$). Afterwards, the motions of the submerged moored body driven by incident waves can be obtained. With the motion results, the moored body moves to a new position, meaning the wave–structure interaction in this time step is finished, and the program is ready for the next time step.

## Model verification and discussions

Two available laboratory tests^22,24^ are adopted to validate the capability of the proposed model. The experiments are carried out in a two-dimensional wave tank at Nagoya University, and Fig. 2 gives the schematic diagram of the numerical set-up. The floating body is anchored to the bottom of the tank with mooring chains, and the inclinations of mooring lines are 90° in the first experiment^22^ and 60° in the second experiment^24^. Figure 4 outlines two instantaneous positions of the vertically moored submerged floating body constrained by mooring lines during its interaction with incident wave, indicating the floating body is free to heave and sway. Figure 5 also outlines two instantaneous positions of the inclinedly moored submerged floating body, indicating the floating body is free to heave, sway and pitch. The detailed hydrodynamic parameters of two experimental cases are shown in Table 1, which are utilized to validate the numerical model by comparing mooring forces and motions of the floating body with the experimental data.

**Table 1:** Experimental cases.

Case	Inclined angle ${\beta }_{0}$	Mass *M* (kg)	Width *B* $(\mathrm{m})$	Thickness *H* $(\mathrm{m})$	Water depth $D (\mathrm{cm})$	Submerged depth $d (\mathrm{cm})$	Wave period ${T}_{i} (\mathrm{s})$	Wave height ${H}_{i} (\mathrm{cm})$
1	90	18.7	0.304	0.137	65	3	1.3	7.7
2	60	28.6	0.4	0.15	60	10.2	1.0	4.6

The weight of the floating is assumed slight compared with the buoyancy force acting on it so that the mooring lines always remain in tension during the interaction between the moored body and the incident surface wave, which avoids the impulsive force acting on the mooring chains. If the mooring chains become slack, the impulsive force develops on them and it may cause the failure of the mooring lines easily. Moreover, the slacking of the mooring lines causes the dynamic movement of the moored body more complex. For details about the experimental set-up, the reader is referred to Rahman et al*.*^22^ and Peng et al*.*^24^.

In the numerical model, model sensitivity tests have been conducted with different grid resolutions and courant numbers related to the time step. In the background mesh, we finally chose *dx* = wavelength/80 and *dy* = wave height/15 for the near water surface zone as the mesh resolution in background mesh. In the component mesh, we finally chose *dx* = minimum (wavelength/160, *B*/30) and *dy* = minimum (wave height/25, *h*/20) for the zone near the floating body. The courant number is chosen as 0.1 in the whole computational period.

### Motion responses of vertically moored floating body

**Figure 7:**
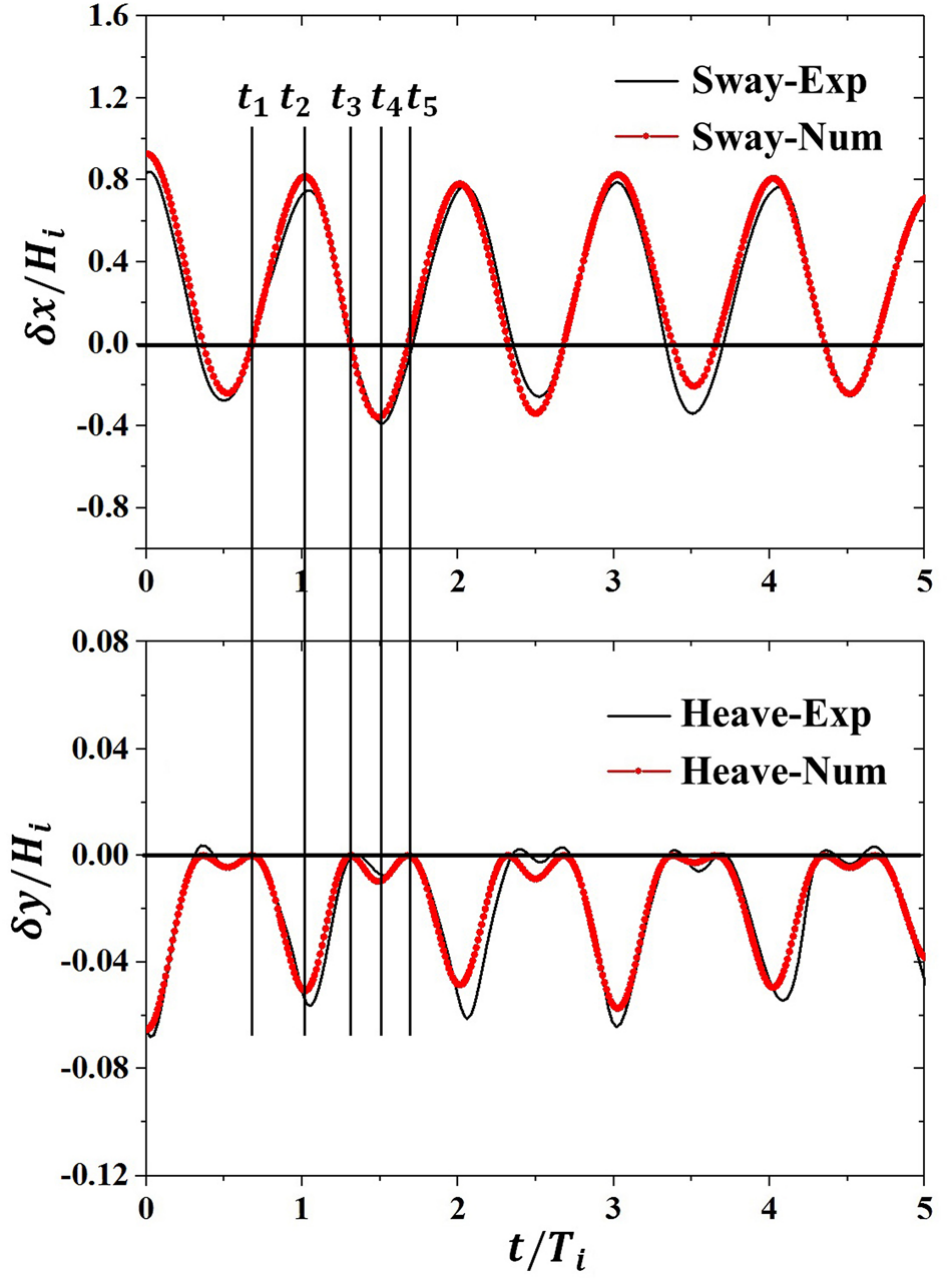
Motion responses of vertically moored floating body. *Exp* experimental data^22^, *Num* numerical results using current model.

In the case of the mooring lines anchored vertically, there occurs no pitch displacement of the body and only the sway and heave values are gain in the wave–structure interaction. Figure 7 presents the time series of numerical results about motion responses of the vertically moored floating body in the current proposed model, which are verified with measured results from the experimental data in Case 1. The results of the comparison in Fig. 7 are plotted as a function of dimensional time for five wave periods, and a good agreement of the numerical simulation results compared to measured data is gained, revealing that developed numerical model can give an accurate prediction of the fierce interaction between vertically moored floating body with wave. The dimensionless numbers, $\delta x/{H}_{i}$ and $\delta y/{H}_{i}$, respectively represent the instantaneous sway and heave displacements.

The positive sway magnitudes represent horizontal displacements of the floating body along the direction of the positive *x*-axis. On the other hand, the negative sway magnitudes represent horizontal displacements of the floating body along the direction of the negative *x*-axis. The positive heave values represent its displacement along the upward direction and the negative heave values represent its displacement along the downward direction. In Fig. 7, it’s seen that sway magnitudes along the onshore direction are about two times as large as that along the offshore direction, indicating the onshore movement of vertically moored floating body driven by the incident surface wave is fiercer than offshore movement. Figure 7 also shows that no positive heave displacement appears in the monitoring time series, indicating the center of the moored floating body stays at its maximum height in the initial state and the mooring chains are incapable of being stretched when the body moves.

To distinguish the results more clearly, five lines are drawn to mark different time instants in one period, namely, *t*_1_–*t*_5_. At *t*_1_ stage, the moored body stays in its initial place, and both sway motion and heave motion equal to zero. Subsequently, the body moves in onshore direction under external wave forces and mooring forces before it reaches its maximum sway displacement at *t*_2_ stage, where the heave displacement also reaches maximum. The body departs from the maximum displacements point and moves back to its initial place at the states between *t*_2_ and *t*_3_. Time series of sway motion in the second half wave cycle from *t*_3_ to *t*_5_ are similar to that in the first half wave cycle from *t*_1_ to *t*_3_. Two pairs of peak and valley values for the heave motion are observed in Fig. 7, which is attributed to the fact that the mooring chains are incapable of being stretched and no positive heave displacement is allowed in the wave–structure interaction. It is also noteworthy that the first half wave cycle from *t*_1_ to *t*_3_ is longer than the second half wave cycle from *t*_3_ to *t*_5_, implying the vertically moored floating body experiences a longer onshore movement than offshore movement, which is closely correlated with the strongly nonlinear and fierce wave–structure interaction.

**Figure 8:**
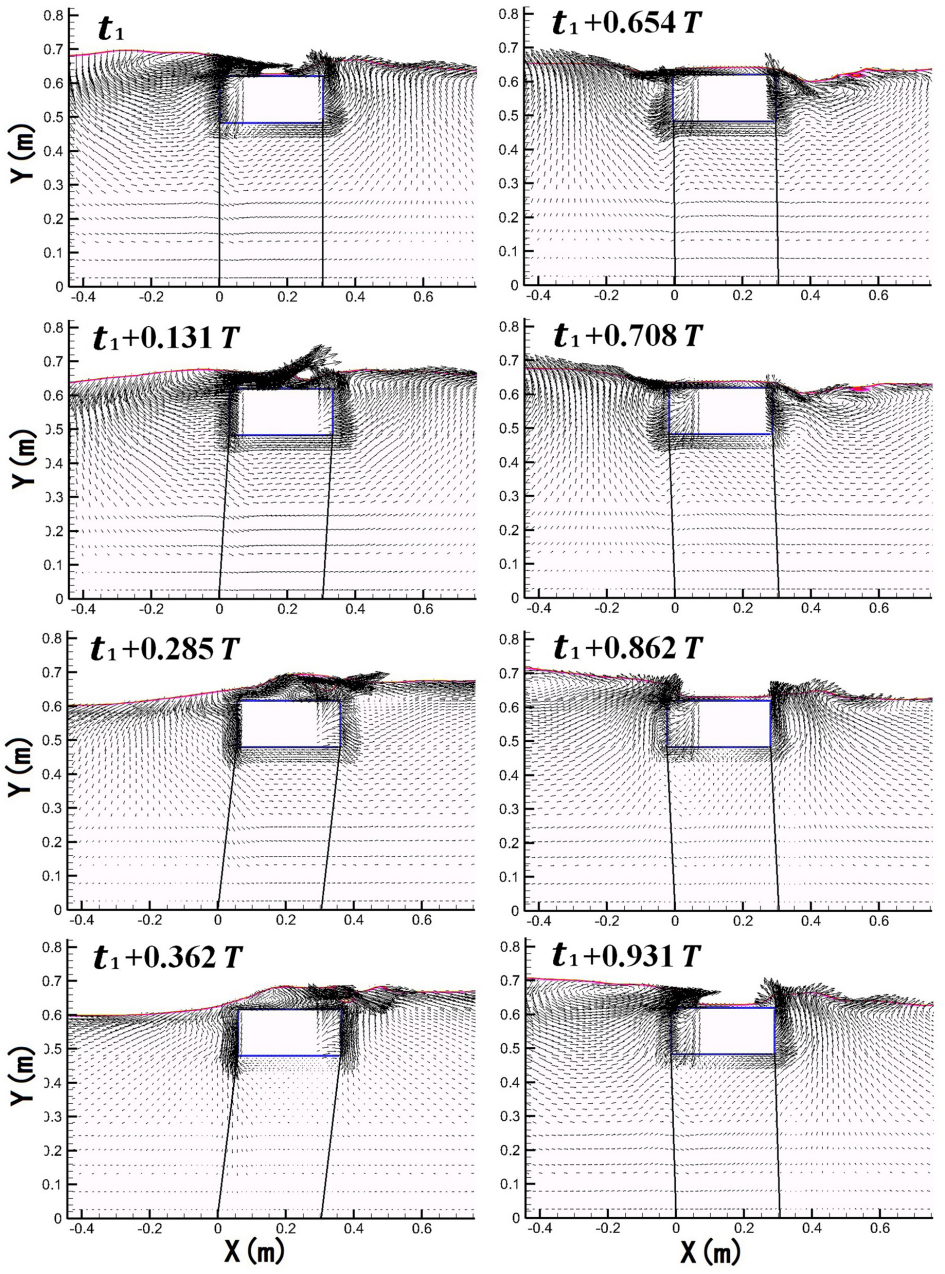
Instantaneous water surface profiles and velocity vectors around the vertically moored floating body in one wave cycle.

In order to illustrate the details of the wave–structure interaction, instantaneous water surface profiles, and velocity vectors around the vertically moored body for different stages in one wave cycle is presented in Fig. 8. The positions of the moored body shown in the figure are set by the numerical results of the sway and heave displacements of the body. The moored body stays in its initial place at *t*_1_ when no motion responses of the body happen. In the figure, it is seen that the wavefront is passing over the floating body and the body moves in the onshore direction due to wave forces from *t* = 0–0.285* T*. The body then gradually moves to the reverse direction and comes back to its initial position at around *t* = 0.654* T*. After that, the floating body moves to the offshore side and returns to its initial position from *t* = 0.708* T*–0.931* T*. Vortex first appears on the top of the body between *t* = 0.285* T*–0.362* T*, and disappears at *t* = 0.654* T*, which is attributed to the wave overtopping and wave breaking on the top of the body. It is also observed that vortex appears around the sharp corners of the floating body at other instants, while the phenomenon is not obvious, which is probably attributed to no pitch motion of vertically moored floating body happens during wave–structure interaction. The magnitude and direction of the water particle velocities on the onshore side change abruptly near the free surface compared with that on the offshore side, which is caused by the fact that the wave energy is partly dissipated and reflected by the floating body, and transmitted wave energy forms secondary wave crest in the rear area of the body.

### Motion responses of inclinedly moored floating body

**Figure 9:**
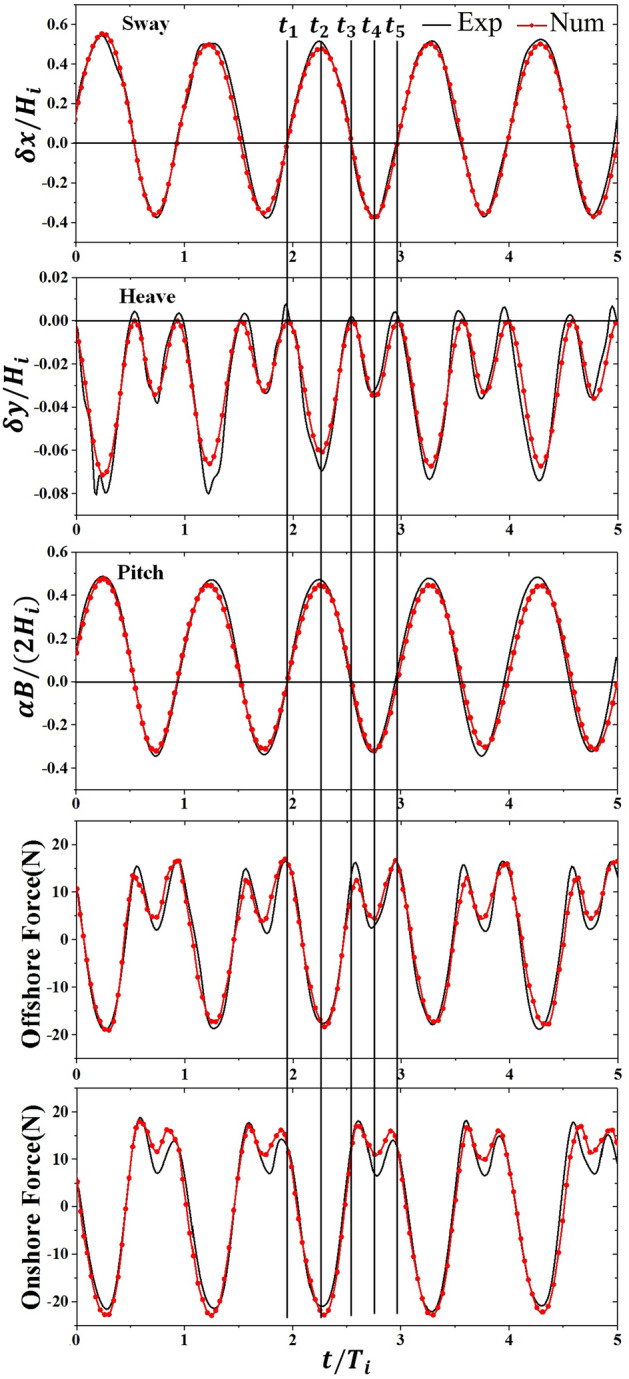
Motion responses of inclinedly moored floating body. *Exp* experimental datayy^24^, *Num* numerical results using current model.

Time series of numerical results about the dynamic motions of the inclinedly moored floating body as well as mooring forces acting on it are verified with measured results from the experimental data in Case 2, which is presented in Fig. 9. The dimensionless number, $\alpha B/(2{H}_{\mathrm{i}})$, represents the instantaneous pitch displacement. A good agreement of the numerical simulation results compared to measured data is gained, revealing that the developed numerical model can give an accurate prediction of the fierce interaction between inclinedly moored floating body with the incident surface wave. Positive pitch motion represents the anticlockwise rotation of the body, and vice versa for negative pitch motion. The initial mooring forces around 34.51 N on each mooring chain in the condition of still water level are removed in Fig. 9, and the positive force represents a larger force than the initial force, and vice versa for negative force. No slack state occurs in the mooring lines during the process of wave–structure interaction as the magnitude of negative tensile forces presented in Fig. 9 are always smaller than initial forces.

To distinguish the results more clearly, five lines are drawn to mark different time instants in one period, namely, *t*_1_–*t*_5_. At *t*_0_ stage, the moored body stays in its initial place, and all motion responses, including sway motion, pitch motion and heave motion, equal to zero, while the peak values of mooring forces have slight phases difference with motion responses, especially onshore mooring force. The floating body at *t*_1_ stage is quickly changing its position as the motion velocities calculated from motion time series are largest in a wave cycle. At *t*_2_ stage, three motion responses reach peak values at the same time, and no phase difference is found in the time series of mooring forces as they all reach valley values. As the body departs from the maximum displacements point, the mooring forces increase to the peak value again at an instant close to *t*_3_ stage. Time series of body motions except for heave motion in the second half wave cycle from *t*_3_ to *t*_5_ are similar to that in the first half wave cycle from *t*_1_ to *t*_3_. For mooring forces, the valley values happen at the stage when the moored body is in the largest motion response, while the peak values appear at the instants close to the initial position.

It should also be noted that two pairs of peak and valley values for the mooring forces and heave motion are observed in Fig. 9, while sway and heave motions only have one peak and valley value, which is similar to what is found in the observations of a vertically moored floating body. This phenomenon is correlated with the constraint that no positive heave value and inversed tensile force is allowed in the interaction. Similar to what is found in the case of a vertically moored floating body, the first half wave cycle from *t*_1_ to *t*_3_ is longer than the second half wave cycle from *t*_3_ to *t*_5_, which is attributed to the strongly nonlinear and fierce wave–structure interaction.

**Figure 10:**
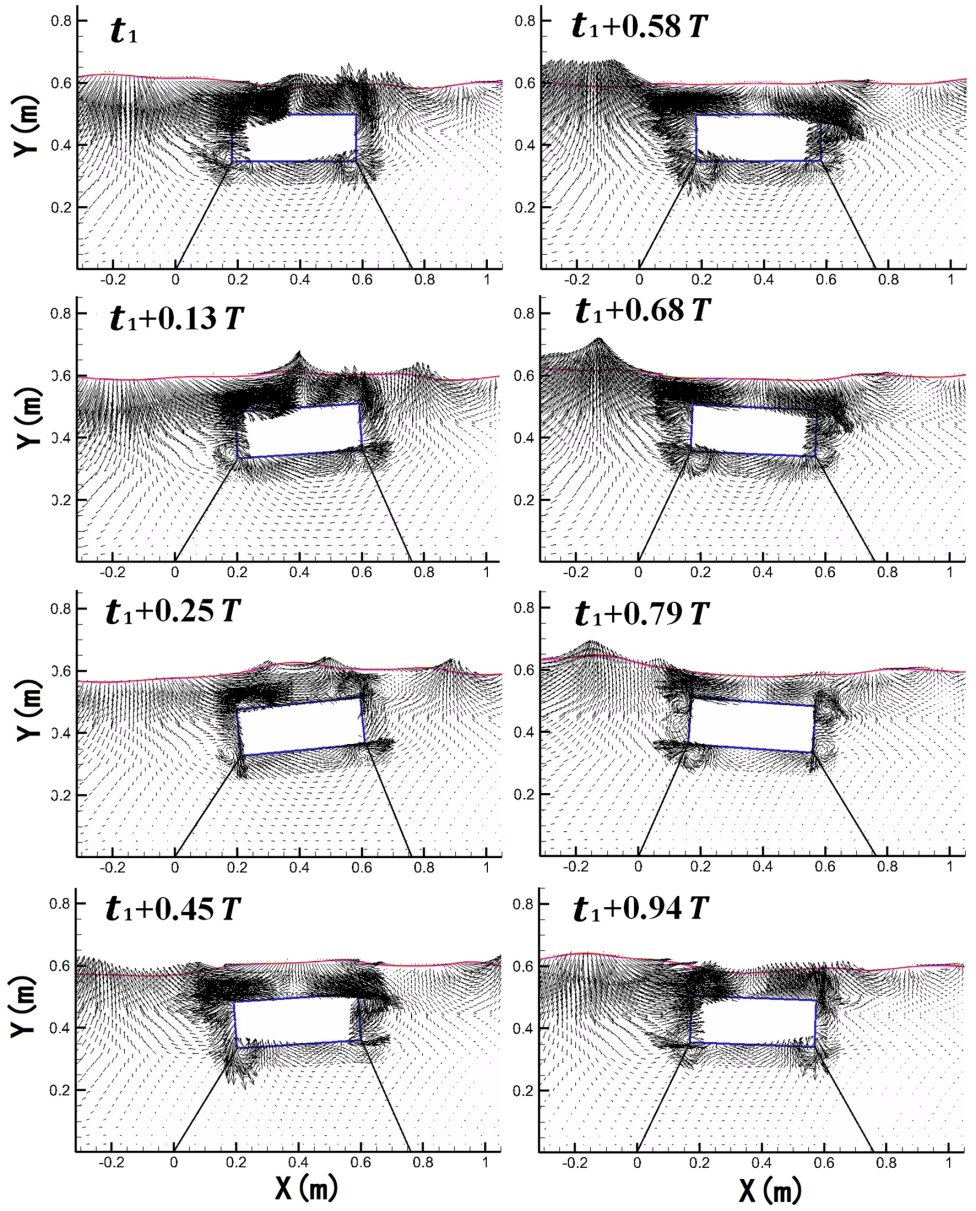
Instantaneous water surface profiles and velocity vectors around the inclinedly moored floating body in one wave cycle.

In order to illustrate the details of the wave–structure interaction, instantaneous water surface profiles, and velocity vectors around the inclinedly moored body for different stages in one wave cycle is presented in Fig. 10. In the beginning, the moored body is in an initial position with nearly zero motion displacement. Driven by hydrodynamic force caused by the periodical wave surface, the body rotates counterclockwise and moves to the onshore side constrained by the mooring lines at *t* = 0.13* T* to 0.25* T*. After that, the body gradually moves in the reverse direction and comes back to its initial position at around *t* = 0.58* T*, and the movement in the first half wave cycle is finished. The offshore movement in second half wave cycle is similar to the motion in the first half wave cycle except that the duration of the second half wave cycle is smaller, demonstrating once again that the onshore movement lasts longer than offshore movement in each wave period.

The details of velocity field around the body are also illustrated in Fig. 10. As wave energy is partly dissipated and reflected by the moving body, the velocity field on the onshore side of the floating body is relatively calm compared with that on the offshore side. Different from what is observed in the velocity field around vertically moored floating body, the phenomenon of vortex generation and dissipation around the four sharp corners of is clearly observed, which is induced by the combined effect of the development of a boundary layer around the solid surface and the nonlinear interaction of the free wave surface with the obstacle, where pitch motion plays a critically important role. It should be noted that the reason why vortex generates more easily around the corner of the inclinedly moored structure than vertical moored structure is that velocities at the corner are relatively large when the inclinedly moored body rotates around its gravity center, and flow separation is easily caused by viscous effect at the corner, which has been demonstrated in Jung et al*.*^27^. It is also noteworthy that rotation directions of the vortexes do not always follow the rotate motion of the body, and phase lags exist between them. Overall, the proposed model gives a good prediction of the velocity field around the body.

## Hydrodynamic response of a submerged floating tunnel

**Figure 11:**
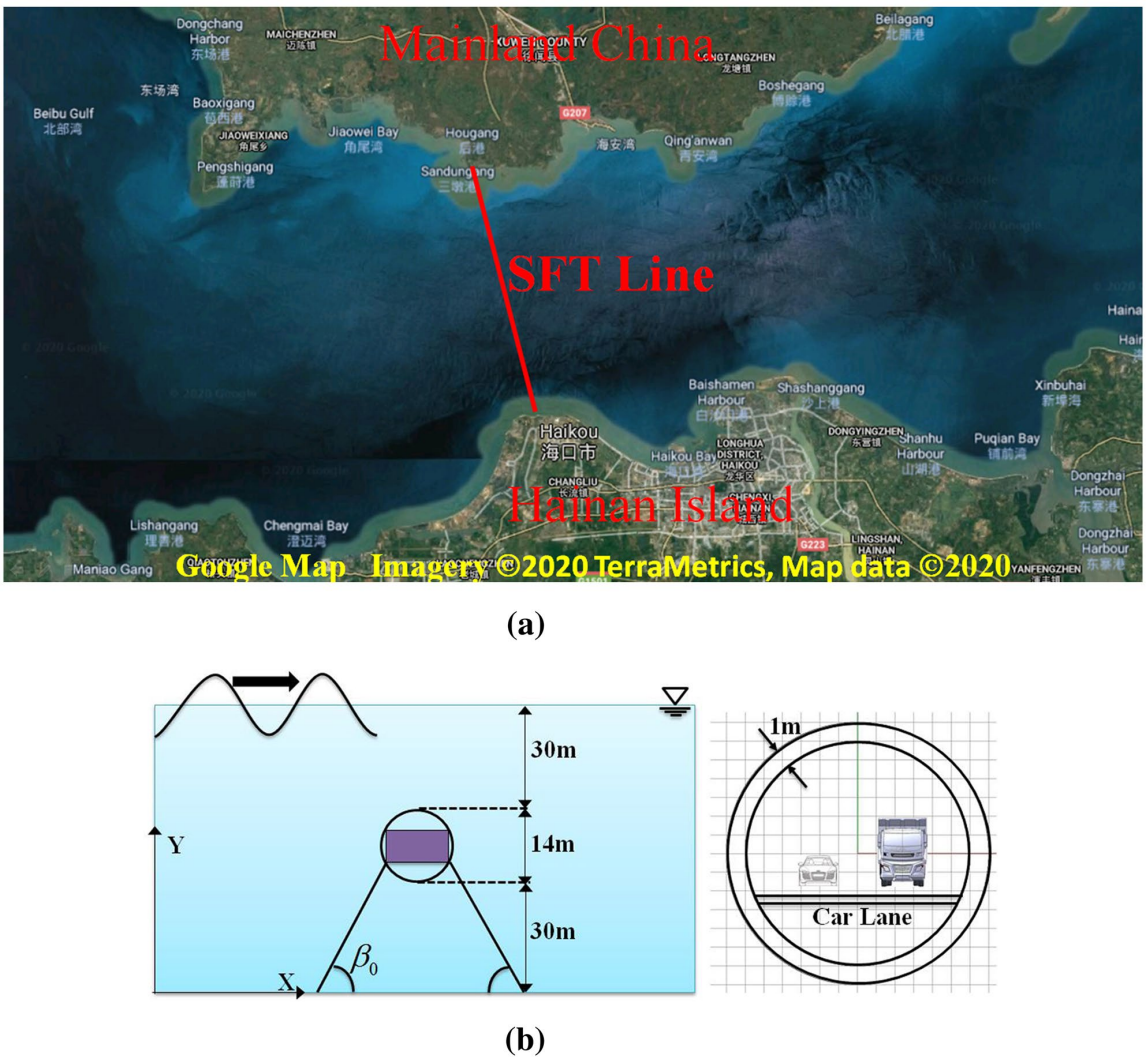
Submerged floating tunnel designed in Qiongzhou Strait, China. (**a**) Qiongzhou Strait (Google Map, https://www.google.com/maps/@20.1563094,110.247553,63900m/data=!3m1!1e3); (**b**) parameters of structure and local water condition.

Recently, Hainan Free Trade Zone in China has been established to further promote the local economic development, while traditional transportation method, i.e., ship, aircraft, is getting more and more difficult to satisfy the rapidly increased demand of transportation between Mainland China and Hainan Island. As seen in Fig. 11a, Qiongzhou Strait is located in the south of China, between Mainland China and Hainan Island, and the crossing of Qiongzhou Strait has been widely discussed by researchers and engineers. Compared with a bridge or a submarine tunnel, SFT is considered to be a better solution for the Qiongzhou Strait waterway crossing due to its distinctive advantages, such as shorter distance, lower cost, less impact on environment and navigation^4,15,52^.

Qiongzhou Strait is one of the largest three straits in China. The length is about 80 km from east to west, and the width ranges from 18 to 35.5 km (29.5 km on average) from north to south. The marked line in Fig. 11a, also known as Central Line, is a good choice among all the proposed lines for the crossing of Qiongzhou Strait as the waterway crossing distance is short, which means shorter travel time and lower operation cost. The average water depth in Central Line is over 50 m with a maximum water depth of 88 m^15^. Figure 11b presents a conceptual design of SFT for Qiongzhou Strait with a constant water depth of 74 m. The Qiongzhou Strait is a busy shipping way. In order to satisfy the requirement of navigation space for big vessels and reduce the impact from the water surface wave, the clearance depth between the water surface and the submerged floating tunnel is proposed to be 30 m^15,52^.

Inclined mooring angle (IMA), which is the initial angle between SFT tether and *x*-axis when SFT is at its equilibrium position, has a great influence on the constraint of SFT, and it is regarded as a key parameter of SFT^2,10^. Hence, to have a better understanding of the motion responses of prototype submerged floating tunnel under waves, we employ the numerical model validated in previous sections to study the influence of IMA on the prototype SFT in Qiongzhou Strait. According to local environmental conditions of Qiongzhou Strait, the wave height with a 25-year return period is 7.1 m, and the corresponding average wave period is 8.7 s, and for a 100-year return period, the wave height and wave period are 8.6 m and 9.6 s, respectively^53^. Taking these extreme wave conditions as a reference, 18 cases are designed for the numerical simulation as shown in Table 2.

**Table 2:** Investigated cases.

Case	Diameter *DD* $(\mathrm{m})$	Water depth $D(\mathrm{m})$	Submerged depth $d(\mathrm{m})$	Concrete Density $\rho (kg/{m}^{3})$	Wave period ${T}_{i} (\mathrm{s})$	Wave height ${H}_{i} (\mathrm{m})$	Inclined angle ${\beta }_{0}$
1	14	74	30	2500	8.7	7.1	25
2	14	74	30	2500	8.7	7.1	35
3	14	74	30	2500	8.7	7.1	45
4	14	74	30	2500	8.7	7.1	50
5	14	74	30	2500	8.7	7.1	53
6	14	74	30	2500	8.7	7.1	60
7	14	74	30	2500	8.7	7.1	67
8	14	74	30	2500	8.7	7.1	75
9	14	74	30	2500	8.7	7.1	90
10	14	74	30	2500	9.6	8.6	25
11	14	74	30	2500	9.6	8.6	35
12	14	74	30	2500	9.6	8.6	45
13	14	74	30	2500	9.6	8.6	50
14	14	74	30	2500	9.6	8.6	53
15	14	74	30	2500	9.6	8.6	60
16	14	74	30	2500	9.6	8.6	67
17	14	74	30	2500	9.6	8.6	75
18	14	74	30	2500	9.6	8.6	90

**Figure 12:**
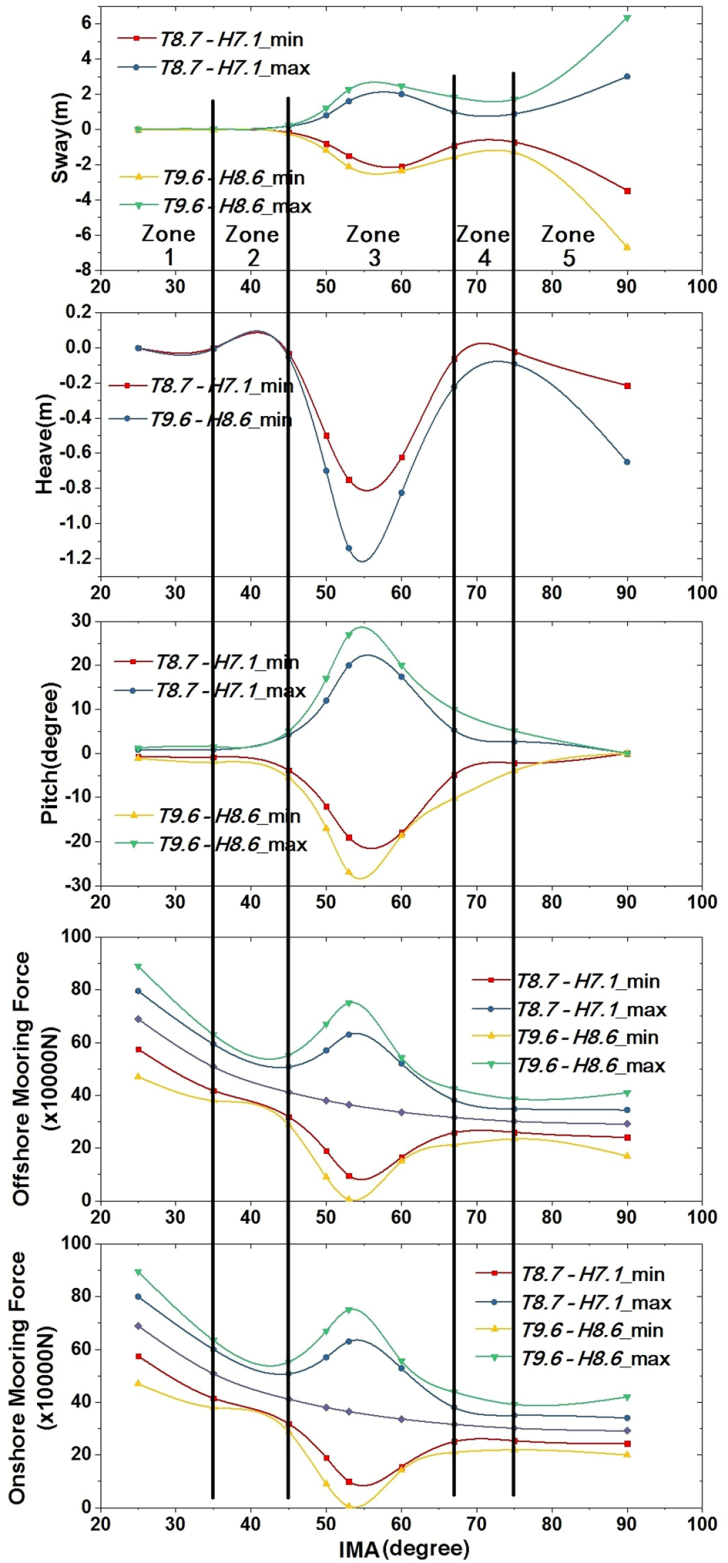
Motion responses of SFT designed in Qiongzhou Strait, China.

The simulated results of mooring forces as well as motion responses with IMA varying in the range of 25°–90° under two extreme wave conditions are presented in Figs.12. It is seen that sway displacements are very small when IMA is less than 45°. After that, sway displacements increase with IMA and reach peak values when IMA is close to 60°. As IMA continues increasing, sway displacements decrease first and then increase to big values larger than the first peak values with increasing IMA. The correlation between heave displacement and IMA is similar to sway displacement except that the heave displacements of the vertically moored floating tunnel are smaller than the first peak values appearing at the range of IMA = 53°–60°. It is also seen that the pitch angle first increases and then decreases with increasing IMA, showing peak values only once, which is slightly different from the observations in the subfigures of sway and heave motions.

The ordinate values in the subfigures of mooring forces in Fig. 12 represent the magnitude of the mooring forces acting on SFT per unit length. Overall, both initial onshore mooring forces and offshore mooring forces in static water decreases with an increase in IMA, which is attributed to the variation of the vertical component of mooring force with different IMA. In the condition of wave–tunnel interaction, the correlation between the mooring forces and IMA is not simply linear or quadratic. Mooring forces decrease with an increase in IMA first before reaching a low point around IMA = 45°, and increase with IMA after that before reaching a peak point at around IMA = 53°. When IMA exceeds 53°, mooring forces decrease with the increase of IMA.

To distinguish the results more clearly, four lines are drawn to separate the range of IMA into five zones as shown in Fig. 12, i.e., Zone 1, Zone 2, Zone 3, Zone 4 and Zone 5. When IMA is smaller than 35° in Zone 1, the mooring forces acting on the floating tunnel are very large, and the corresponding values of three body motions are all at a very low level, indicating the motion responses of SFT designed in Qiongzhou Strait in Zone 1 are safe under the severe wave conditions for 25-year return period and 100-year return period. However, the mooring chains of SFT designed in Zone 1 has a potential to be damaged running the risk of exceeding ultimate tensile strength at extremely high mooring forces, and the reliability of anchor system should be guaranteed for safety. In Zone 2, the motion responses slightly increase while the mooring forces decrease apparently in the range of IMA = 35°–45°. As motion responses in Zone 2 are relatively small and mooring forces are not very large, Zone 2 is considered to be a good choice for the design of IMA in current model. In Zone 3, motion responses all reach peak values, indicating the designed SFT undergo a fierce movement in this condition and the moving road surface in the tunnel can seriously affect the safety of the vehicles and raise the possibility of traffic accidents. Both motion displacements and mooring forces reduce to a certain level in Zone 4. The motion displacements in Zone 4 are larger than that in Zone 2, while the mooring forces in Zone 4 are smaller than that in Zone 2, inferring Zone 4 may also be a good choice for the design of IMA. However, the range of Zone 4 is narrower than Zone2. As IMA continues increasing in Zone 5, sway and heave displacements become larger and larger, while pitch motion reduces to zero when IMA = 90°. The mooring forces in Zone 5 are smallest in all zones. However, as sway and heave motions are fierce, Zone 5 is unacceptable for the design of IMA.

As seen in Fig. 12, motion responses show a slight difference between severe wave conditions for 25-year return period and 100-year return period in Zone 1 and Zone 2, while significant differences are found in Zone 3, Zone 4 and Zone 5. This finding infers again Zone 2 is a good choice for the design of IMA, and Zone 1 may also be recommended as long as the higher requirements for the strengths of mooring chains and anchoring are satisfied. Given the motion responses and mooring forces in Zone 4 are not large in the wave condition for 100-year return period, it is still regarded as an option for the choice of IMA. It’s also noteworthy that the minimum mooring forces at around IMA = 53° within Zone 3 are close to zero under the wave condition of 100-year return period, inferring the mooring chains has a potential to go slack, which probably leads to a sudden large increase in the maximum mooring force. The sudden increases of mooring force may be a maximum of around 20 times as large as normal forces in the mooring chains without slack^10^.

Figure 12 gives detailed motion responses of SFT within different IMA designed in Qiongzhou Strait, and the findings provide guidance for the choice of IMA in engineering design. Overall, Zone 2 is considered to be the best choice for the design of IMA for both motion displacements and mooring forces are relatively small in this zone, and Zone 1 is also a good choice as long as the reliability of anchor system is guaranteed. Though motion displacements in Zone 4 reach a medium level under severe wave conditions, Zone 4 may also be an option for the choice of IMA as the corresponding mooring forces are relatively small. The SFT in Zone 5 experiences fierce movements and the safety of the vehicles passing through the tunnel can not be ensured. The design of IMA in Zone 3 is the worst choice as not only are motion responses of SFT severe, but also the mooring chains are at the risk of going slack under severe wave conditions.

For the study of dynamic plastic response of beams and plates subjected to uniformly distributed pressure, Zhao^54^ proposed a dimensionless number for the dynamic plastic response of beams and plates, which reflected three aspects, i.e., the inertia of the distributed loading, the resistance ability of the material to the deformation and the geometrical influence of the structure. The dimensionless number, termed as response number, is defined by the following equations.

19 $$ R_{n} = D_{n} \left( {\frac{{L^{\prime}}}{{H^{\prime}}}} \right)^{2} $$

20 $$ D_{{\text{n}}} = \frac{{I_{e}^{2} }}{{\rho \sigma_{0} H^{^{\prime}2} }} $$

21 $$ I_{e} = \mathop \smallint \limits_{{t_{0} }}^{{t_{f} }} p\left( t \right)dt $$

where $$R_{n}$$ and $$D_{n} $$ are the response number and damage number, respectively. $$H^{\prime}$$ and $$L^{\prime} $$ are taken to be the thickness and semi-length of the structure, respectively. $$\sigma_{0}$$ is the resistance ability of the material and $$I_{e}$$ is the effective impulse of the distributed loading.$$ t_{0}$$ and $$t_{f}$$ are the moments when deformation begins and ends.$$ p$$ $$\left(t\right)$$ is the distributed pressure.

The boundary condition of the freely floating tunnel under wave action is free-free, and the plastic displacement, $w_{f}$, can be calculated by the following equation.

22 $$ \frac{{w_{f} }}{{H^{\prime}}} = \frac{Rn}{6}\left( {1 - \frac{{p_{0} }}{p}} \right) $$

When $\frac{{p_{0} }}{p} \to 0,$ the limit of Eq. (20) can be expressed as

23 $$ \frac{{w_{f} }}{H} = \frac{Rn}{6} $$

**Figure 13:**
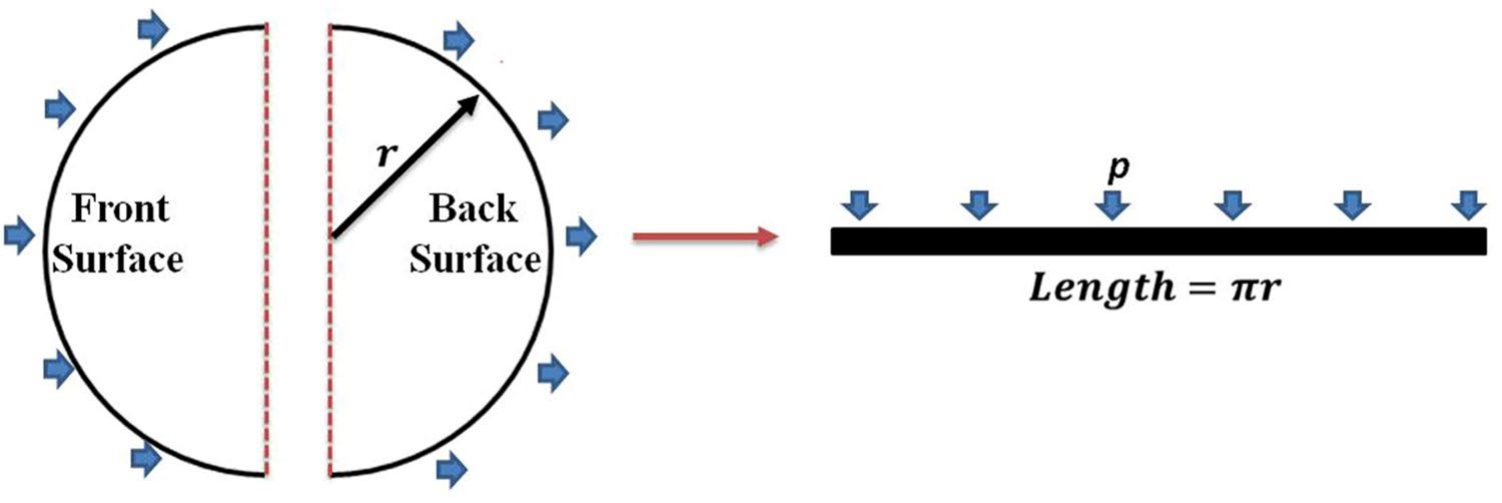
Assumption of the model in the plastic response.

**Figure 14:**
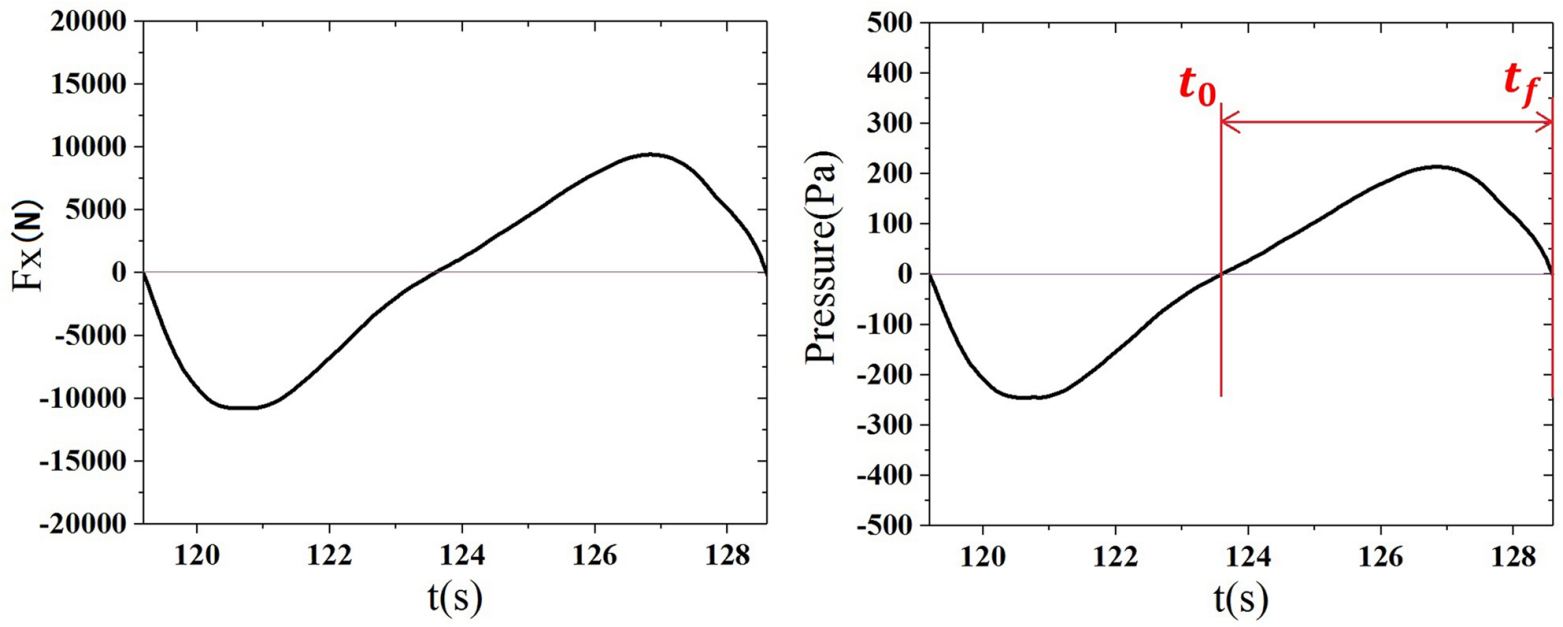
Resultant horizontal force and the corresponding net pressure acting on the SFT in a wave period of Case 12 within Zone 2.

The distributed net pressure around the tunnel surface is nonuniform. To get an approximation of the deformation of the tunnel under wave action, the distributed net pressure is assumed to be uniform, which is proportional to the resultant forces in both horizontal and vertical directions. As the amplitudes of sway motion are much larger than heave motion shown in Fig. 12, the net horizontal forces in wave action are utilized to calculate the uniformly distributed pressure. As mentioned above, Zone 1 and Zone 2 are considered to be good choices for the design of IMA, while Zone 3 is the worst choice. The deformations of SFT in Case 11, Case 12 and Case 15 located in Zone 1, Zone 2 and Zone 3 are respectively calculated. As the circle surface is symmetry, the length of the structure in the plastic response is assumed to be half of the perimeter as shown in Fig. 13. Figure 14 presents the resultant horizontal force and the corresponding net pressure acting on the SFT in a wave period of Case 12 located in Zone 2. It’s seen that the value of $I_{e}$ in Eq. (19) is very small if ${t}_{f}-{t}_{0}$ equals to a wave period as the curves of force and net pressure are nearly symmetric within one wave period. To estimate the maximum deformation, ${t}_{f}-{t}_{0}$ equals to ${T}_{i}/2$. The resistance ability of the reinforced concrete, ${\sigma }_{0}$, is assumed to be 40 MPa.

Based on Eq. (21), the limits of plastic displacement in Case 11, Case 12 and Case 15 are calculated, and the results are 0.0000064 m, 0.0032 m and 0.086 m, respectively. The maximum deformations in the suggested Zone 1 and Zone 2 are negligible compared with the diameter of the SFT, indicating the choice of Zone 1 and Zone 2 is applicable in the engineering when the deformation is considered. Although the deformation in Zone 3 reaches a certain value, it is still reasonable to use a rigid model to calculate the motion responses of SFT as the relative deformation,${\mathrm{w}}_{f}/D$, is 0.61%, which is small compared with the length and motion displacements. To reduce the maximum deformation in the engineering application, an increase of thickness is taken as an efficient way according to Eqs. (17–21). Besides, the choice of new reinforced concrete with higher resistance ability is also a good way to reduce the maximum deformation according to Eq. (18).

## Summary and conclusions

In this paper, a numerical model for studying the motion responses of vertically and inclinedly moored SFT with multiple degrees of freedom under wave forces is proposed. The N–S equations are used as the governing equations to describe the flow field. In the numerical wave tank, two wave absorption zones are distributed in the front and rear of the SFT to absorb reflected waves and transmitted waves, making sure the working zone is not affected by reflected waves. A description of the methodology focusing on the mechanics of the vertically and inclinedly moored floating body is presented in this paper. To preserve the mesh quality around the body during the entire computation, overset meshing methodology is utilized in the numerical simulation and high mesh quality is preserved in the process of fierce fluid–structure interaction.

Two laboratory experiments are used for validating the numerical model focusing on a vertically moored floating body and inclinedly moored floating body, respectively. A good agreement is gained by comparing the simulated results with measured data in terms of motion responses and mooring forces, indicating the proposed model is capable of simulating the interactions between surface waves and SFT constrained by mooring chains. It is found in the results that the onshore movement in the first half wave cycle from lasts longer than offshore movement in the second half wave cycle for both vertically and inclinedly moored body in each wave period, implying the interaction is strongly nonlinear and the body experiences fiercer motions along the direction of wave propagation. It is also found that vortex generates more easily around the corner of the inclinedly moored structure than the vertical moored structure, which is because the velocities at the corner are relatively large when the inclinedly moored body rotates around its gravity center, and flow separation is easily caused by viscous effect at the corner.

The prototype SFT under severe wave conditions of a 25-year return period and 100-year return period designed for Qiongzhou Strait located between Mainland China and Hainan Island has been simulated using the verified hydrodynamic model for wave–structure interaction. The numerical simulations shed light on the offshore and onshore mooring forces, as well as pitch, sway and heave responses of the SFT under different IMA conditions. It’s seen that peak values exist in three motion responses, and the corresponding mooring forces also reach to local maximums and minimums. Large mooring forces means a high requirement for the strengths of mooring chains and anchoring, and small mooring forces may also bring damage to the mooring system for the possibility of the slack phenomenon. To provide guidance for the design of IMA, the range of IMA is separated into five zones. Zone 2 is regarded as the best choice for the design of IMA for both motion displacements and mooring forces are small in this zone, while Zone 3 is considered to be the worst choice as not only are motion responses of SFT severe in this zone, but also the mooring chains are at the risk of going slack under severe wave conditions.
